# Optimization of pharmacological interventions in the guinea pig animal model—a new approach to calculate the perilymph volume of the scala tympani

**DOI:** 10.3389/fnins.2023.1297046

**Published:** 2023-12-15

**Authors:** Marleen Grzybowski, Kathrin Malfeld, Thomas Lenarz, Verena Scheper, Daniel Schurzig

**Affiliations:** ^1^Department of Otorhinolaryngology, Head and Neck Surgery, Hannover Medical School, Hannover, Germany; ^2^German Hearing Center Hannover, Hannover Medical School, Hannover, Germany; ^3^Center for Biomedical Engineering, Implant Research and Development (NIFE), Hannover Medical School, Hannover, Germany; ^4^MED-EL Research Center, Hannover, Germany

**Keywords:** cochlear modeling, individualized implantation, pharmacokinetics, drug delivery, cochlear implantation, electrode coating

## Abstract

**Objective:**

The guinea pig serves as a well-established animal model for inner ear research, offering valuable insights into the anatomy, physiology, and therapeutic interventions of the auditory system. However, the heterogeneity of results observed in both *in-vivo* experiments and clinical studies poses challenges in understanding and optimizing pharmacotherapy outcomes. This heterogeneity may be due to individual differences in the size of the guinea pig cochlea and thus in the volume of the scala tympani (ST), which can lead to different drug concentrations in the ST, a fact that has been largely overlooked thus far. To address this issue, we aimed to develop an approach for calculating the individual volume of perilymph within the ST before and after cochlear implant insertion.

**Method:**

In this study, high-resolution μCT images of a total of *n* = 42 guinea pig temporal bones were used to determine the volume of the ST. We compared fresh, frozen, and fixed tissues from both colored and albino strains to evaluate the potential influence of tissue condition and strain on the results.

**Results:**

Our findings demonstrate a variability in mean ST volume with a relative standard deviation (RSD) of 14.7%, comparable to studies conducted with humans (range RSD: 5 to 20%). This indicates that the guinea pig cochlea exhibits similar variability to that of the human cochlea. Consequently, it is crucial to consider this variability when designing and conducting studies utilizing the guinea pig as an animal model. Furthermore, we successfully developed a tool capable of estimating ST volume without the need for manual segmentation, employing two geometric parameters, basal diameter (A) and width (B) of the cochlea, corresponding to the cochlear footprint. The tool is available for free download and use on our website.

**Conclusion:**

This novel approach provides researchers with a valuable tool to calculate individual ST volume in guinea pigs, enabling more precise dosing strategies and optimization of drug concentrations for pharmacotherapy studies. Moreover, our study underscores the importance of acknowledging and accounting for inter-individual variability in animal models to enhance the translational relevance and applicability of research outcomes in the field of inner ear investigations.

## Introduction

Cochlear implants (CIs) have been continuously developed over the past decades and are the gold standard for the treatment of sensorineural hearing loss. The CI function is based on bypassing the cochlear sensory cells by direct stimulation of the primary auditory neurons (spiral ganglion neurons, SGNs), leading to hearing sensation. Due to the ongoing development, more patients with an increasing spectrum of sensorineural hearing loss can be treated. It is now common practice to implant patients with good residual hearing in the low frequencies and little to no residual hearing in the middle and high frequencies (Cullen et al., 2004; Ilberg et al., 2011). Nevertheless, insertion depth, length, mechanical characteristics of the CI (Dhanasingh and Jolly, 2017) and the surgical technique may directly lead to implantation related hearing loss (Lenarz and Scheper, 2015). Mechanical injuries of the basilar membrane or osseous spiral lamina, which can affect the endo-cochlear potential, create oxidative stress and initiate pro-apoptotic pathways associated with direct injury to and loss of hair cells (Bas et al., 2012). Next to loss of residual hearing, a fibrotic encapsulation of the CI (Clark et al., 1995; Li et al., 2007) [and degeneration of the SGN (Seyyedi et al., 2014)] may lead to suboptimal CI performance. That is why an increasing number of studies is exploring pharmacotherapies to protect the fine intra-cochlear structures, including their neural connections, from implantation related subsequent indirect tissue damage.

To preserve remaining hair cells from implantation related damage, acute as well as chronic therapies are under investigation. Studies to prevent loss of residual hearing mainly focus on corticosteroids, c-Jun N-terminal kinase (JNK) inhibitors, and antioxidants. An increasing number of studies reports that corticosteroids, such as dexamethasone (DEX), protect against electrode insertion related hearing loss. Using animal models, a recovery of the compound action potential (CAP) or acoustically evoked auditory brainstem response (ABR) threshold following corticosteroid treatment in implanted inner ears was reported (Ye et al., 2007; Eastwood et al., 2010; Braun et al., 2011). However, there are contrary reports as well in which no beneficial effect of corticosteroids on hearing preservation could be found (Stathopoulos et al., 2014; Wilk et al., 2016; Ahmadi et al., 2019). Furthermore, some clinical trials demonstrated a beneficial effect of glucocorticoid administration on residual hearing (Skarzynska et al., 2022) while others failed to do so (Eshraghi et al., 2007; Vivero et al., 2008). The same is true for drug effects on CI fibrotic encapsulation, which can either be directly analyzed in animal models via histology or indirectly investigated in patients via impedance analysis. Unfortunately, corresponding studies found mixed results as well: some groups reported reduced fibrosis and/or impedances by steroid application (Paasche et al., 2006; Farhadi et al., 2013; Prenzler et al., 2018) while others found no effect of drugs onto the aforementioned readouts (Huang et al., 2007; Liu et al., 2015). Finally, SGN regeneration for optimizing CI outcomes is in the focus of pharmacotherapy approaches as well. Here again, study results are heterogeneous with reports on positive (Warnecke et al., 2012; Bas et al., 2019; Chambers et al., 2019; Scheper et al., 2019, 2020) as well as no effects (Huang et al., 2007).

The administration of active components to protect intracochlear structures can be accomplished by a variety of routes. Most commonly in the clinical setting, active components are administered systemically for inner ear disorders (Musazzi et al., 2018). However, a disadvantage of systemic drug administration is that highly concentrated drug amounts may be required to achieve a biologically effective concentration in the inner ear. This can lead to systemic side effects (McCall et al., 2012). To overcome this drawback, drugs can be administered directly into the middle ear cavity, i.e., intratympanically (IT) by injection with a needle through the eardrum. IT administered drugs are absorbed into the inner ear perilymph through the round window membrane and to a smaller extent through the oval window. Bird and colleagues demonstrated 425- to 45,000-fold higher drug levels, corrected for dose bypassing the blood-labyrinth barrier and first-pass metabolism, when comparing drug concentrations in the perilymph after systemic administration and IT injection (Bird et al., 2007, 2011). However, intratympanic administration also has disadvantages as large differences in perilymph concentrations can occur because portions of the drug may flow back when the middle ear is apparently “full.” Partially injected solution may also disappear from the middle ear due to drainage into the Eustachian tube. Furthermore, air bubbles, a false membrane or fat on the round window may prevent drug access to the inner ear (Zhuo et al., 2023). Round window niche implants, inserted after visual inspection and cleaning of the niche, eluting drugs directly at the round window membrane are under development as possible alternative to uncontrolled intratympanic drug application (Matin-Mann et al., 2022, 2023). To circumvent disadvantages of systemic and intratympanic administration, studies are investigating direct delivery of drug into the scala tympani (ST). This requires opening the inner ear, which may lead to side effects such as transient or permanent threshold shifts. Therefore, this delivery approach is currently only used for gene therapy approaches (Staecker et al., 2014; Ramekers et al., 2015; Pfannenstiel et al., 2020; St Peter et al., 2022), which are not yet part of the clinical routine, or in combination with cochlear implantation. In case of the latter, drugs can be injected as a single bolus into the ST followed by insertion of the CI (Paasche et al., 2006; Braun et al., 2011; Prenzler et al., 2018, 2020). In order to achieve a longer time period of drug application, the CI silicone can alternatively be used as a drug depot (Vivero et al., 2008; Farhadi et al., 2013; Ramekers et al., 2015; Wilk et al., 2016; Scheper et al., 2017) or be coated with a matrix releasing the respective drug (Kikkawa et al., 2014; Wrzeszcz et al., 2014; Bas et al., 2019; Scheper et al., 2019).

Before (new) release methods or active substances can be tested in patients, it must first be shown in animal models that they are safe and not ototoxic. The guinea pig is an established animal model for inner ear research, both for studies of the anatomy and physiology of the intact auditory system and for exploring therapeutic options for a wide range of pathologies. However, results from both *in vivo* experiments and clinical studies are heterogeneous. For example, the studies by Parnes et al. (1999) and Bachmann et al. (2001) show strongly different perilymph concentrations with similar applications. These differences may be due to the complexity of pharmacodynamics and pharmacokinetics during drug administration in the inner ear, which is still not fully understood. Salt and colleagues first published a model in 2002, called FluidSim, that can simulate the distribution of drugs in the cochlea for six different species, including humans and guinea pigs (Salt, 2002; Salt, 2023). Since then, this model has been continuously extended (current version Is 4.06), and a wide variety of settings can be made. On the one hand, it is possible to choose how the drug is released (intratympanic application, round window niche implant, intra-cochlear injection, eluting CI, and systemic application), and on the other hand, the drug can be selected from a database so that its molecular properties are considered in the simulation.

As early as 1938, Hardy (1938) showed that human cochlear length has an inter-individual variation of 40%. With increasingly improving imaging modalities and investigation methods, this claim was supported and quantified within various other studies (Avci et al., 2014; Würfel et al., 2014; Meng et al., 2016; Pietsch et al., 2017, 2022; Timm et al., 2018; Schurzig et al., 2018a), showing lateral wall length values of 30–45 mm (Timm et al., 2018) and ST volumes of 23–50 μL (Dhanasingh and Hochmair, 2021). Since these variations also yield very different CI locations inside the inner ear with the same implant array (Weller et al., 2023), these findings are now commonly used within the clinical routine for patient-specific, preoperative cochlear length determination and electrode selection (Timm et al., 2018; Schurzig et al., 2018a; Mertens et al., 2020; Avallone et al., 2021) and even allow for virtual cochlear implantation prior to the actual surgery (Schurzig et al., 2022).

Meanwhile, the individuality of laboratory animals regarding the individual size and shape of the structures and the individual perilymph volume has not been taken into account so far (Salt, 2002; Mynatt et al., 2006; Plontke and Salt, 2006), which may be one factor leading to failure of drug therapy due to insufficient drug dosage. Especially since known values from the literature on guinea pig ST volume range from 4.76 μL (Thorne et al., 1999) to 5.96 μL (Salt, 2023). To obtain information on the precise pharmacokinetics and dynamics of a variety of substances in the inner ear, accurate anatomical dimensions must be used to gain better insight. In addition to the possible individuality of ST volumes, the type of guinea pig and its preparation may also play a role in the inhomogeneity of studies. In this regard, the variability of the studies differs depending on whether colored (Thorne et al., 1999; Plontke et al., 2007; Ye et al., 2007; Scheper et al., 2017) or albino (Parnes et al., 1999; Ramekers et al., 2015; Scheper et al., 2019) animals were used.

The aim of the presented work was to develop an approach to calculate the individual volume of perilymph inside the ST before and after CI insertion in order to allow for calculating optimal concentrations of released drugs, required drug volumes of CIs and required injection volumes to achieve a desired drug concentration in the perilymphatic space. Since the established animal model is the guinea pig, we first created the model for it to test our hypothesis. We used high-resolution μCT images of guinea pig cadaver to determine the ST volume and compared fresh, frozen and fixed tissue of a colored and an albino strain, respectively, to determine whether the tissue condition or strain affects the results.

## Materials and methods

For our volumetric investigation fresh as well as fixed and frozen tissue was used from albino (*n* = 24) and colored (*n* = 18) animals. Both ears per animal were used for analysis. Ten ears of albino guinea pigs and six ears of colored animals were scanned “fresh,” i.e., directly after killing (in the following referenced as Alb. fresh and Col. fresh). Seven albino ears and twelve colored ears were scanned “frozen,” i.e., after storing at −18°C (Alb. frozen and Col. frozen). Eight ears of albino guinea pigs were scanned after long-term storage in 4% paraformaldehyde (PFA), i.e., “fixed” samples (Alb. PFA).

In addition, 15 unilaterally (all left side) cochlear implanted albino guinea pigs were included to derive relation between metric and angular insertion depth. Only the implanted side was included in the analysis.

### Sample preparation

The study was conducted in accordance with the German “Law on Protecting Animals” and with the European Communities Council Directive 2010/63/EU for the protection of animals used for experimental purposes. Three different temporal bone sources were used. All animals were adult and between 2 and 12 months old. Animals of an in-house breeding colony of albino (Dunkin-Hartley) and colored guinea pigs were kindly provided by the working group of Prof. Mazzouli-Weber, Institute for Physiology and Cell Biology, University of Veterinary Medicine Hannover, Hannover. Those animals were sacrificed by bolt shot in combination with throat transection.

Additionally, eight cochleae of adult Dunkin-Hartley guinea pigs, purchased from Charles River Laboratories, France, were used. The use of those animals for scientific purposes was permitted by the regional council (Lower Saxony State Office for Consumer Protection and Food Safety (LAVES), Oldenburg, Germany, under §4, killing animals for tissue sampling). The temporal bones were harvested in all animals of the “fresh” groups after decapitation.

**Figure 1:**
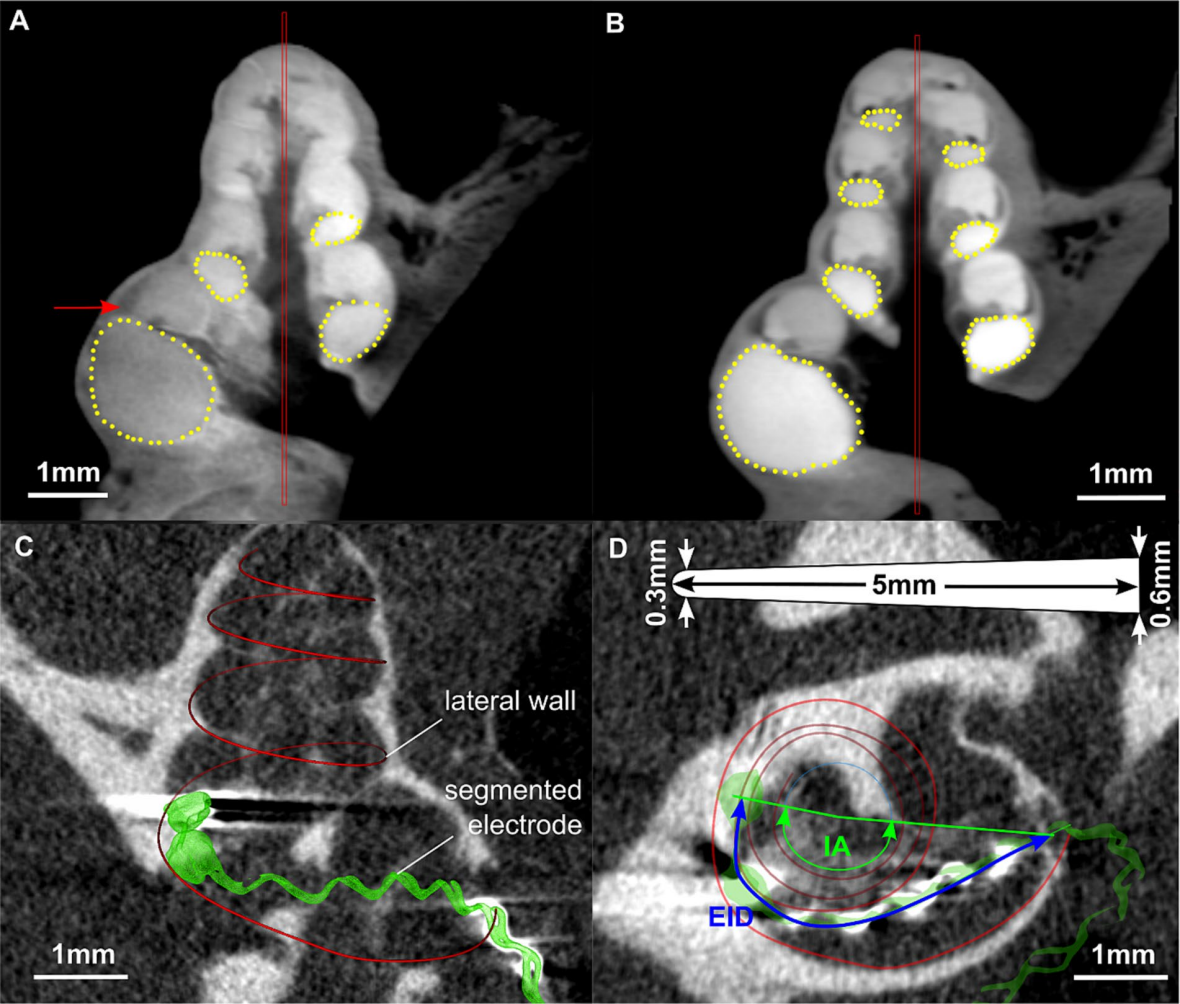
Images of the segmentation of a fresh **(A)** and a frozen **(B)** guinea pig cochlea using COMET (Lexow et al., 2016, 2018). The red bars represent the midmodiolar axis, yellow points indicate the shape of the ST. In **(A)**, the arrow points on the lateral wall tissue which can be well distinguished from the ST. Images **(C)** and **(D)** are from scans performed *in vivo* directly after cochlear implantation. **(C)** Segmentation of lateral wall and electrode array in 3D slicer. **(D)** Corresponding measurement of electrode insertion depth (EID) and insertion angle (IA) in a projected view. The image includes a sketch of the electrode array dimensions, illustrating the length (5 mm), the tip (0.3 mm) and basal (0.6 mm) diameter.

The 15 implanted (non-commercial guinea pig electrode manufactured for this specific purpose, MED-EL, Austria, dimensions depicted in Figure 1D) cochleae from Dunkin-Hartley guinea pigs (Charles River Laboratories, France; regional council registration numbers 21/3703 and 17/2396) were imaged directly after CI insertion to investigate electrode insertion depth and angle. Those images were not used to segment the ST due to the imaging artifacts of the electrode array. Cochlear implanted animals were finally transcardially perfused using PFA but since the analysis was performed on micro-CT scans of living animals, the method of tissue preparation has no impact on the imaging data.

The bulla was opened, and the bony capsule was removed. Using a needle (22G), the cochlea was opened at the apex. The round window membrane was incised using a lancet, and the ST was flushed using Iomeprol (imeron®250, Bracco Imaging Deutschland GmbH, Konstanz, Germany) until the contrast agent rinsed out of the opening at the apex.

Frozen samples were stored at −18°C for a minimum of 6 weeks and thereafter thawed by keeping them at room temperature overnight. The next day, the temporal bones were harvested and processed as described above.

Fixed samples were kept in 4% PFA for a minimum of 6 months and afterwards were processed as described above.

### Imaging

The contrast agent flushed cochleae were placed in an 1 mL Eppendorf tube with the apex pointing to the bottom. The tubes were placed in the carousel of a SCANCO MicroCT 100 [version 1.1, SCANCO Medical AG, Switzerland (Schurzig et al., 2016)]. Samples were scanned at 70kVp, 114 μA and at a voxel size of 30 μm. Afterwards, the region of interest was defined in the reconstruction and transformed into a Digital Image and Communications in Medicine (DICOM) file using the software IPL (ScancoMedical AG).

### Segmentation and measurement

All DICOM datasets containing unimplanted cochleae were manually segmented using the custom research tool “COMET” (Lexow et al., 2016, 2018). The benefit of this software is that after placement of a rotation axis into the center of the modiolus, radial slicing planes are generated which allow for optimal identification and hence segmentation of cochlear structures. For the present project, marker points were placed along the contour of the ST within these midmodiolar slicing planes starting in the center of the round window in angular steps of 22.5 deg. up to the apex. Segmentations were stopped when the imaging quality no longer allowed for clear identification of the ST contour. Depictions of the segmentation procedure in COMET are given in Figures 1A,B. The segmentation points could then be exported in a Matlab compatible file format (.mat) for further processing. The measurement was not blinded but performed by only one person in order to avoid inter-individual differences.

In addition, the DICOM datasets containing implanted cochleae were loaded into 3D Slicer^1^ (Fedorov et al., 2012) to measure the electrode insertion depth (EID) as well as the insertion angle (IA) of the individual cochleae for round window and cochleostomy insertion of a CI. Images showing an example of an implanted cochlea in 3D Slicer and the corresponding measurements are depicted in Figures 1C,D.

### Segmentation processing

All processing of the cross-sectional segmentations was performed in Matlab (version R2018A, Mathworks, USA). Previously validated algorithms were used to extract the basal cochlear diameter (A) and with (B) (Schurzig et al., 2018a) as well as the cochlear volume (Räth et al., 2023, accepted) from all individual segmentations: in brief, for each individual cross section the point with the largest distance to the modiolar wall was computed. The lateral wall of each specimen was then defined as the connecting curve of these points. A and B values were extracted from these lateral wall profiles. Cochlear volume was computed as the sum of volumes in between adjacent cochlear cross sections for each specimen.

### Statistical evaluations

Statistical evaluations were conducted in Python (version 3.7, Python Software Foundation, USA) using the Scipy library (version 1.2.1). Significance of differences in parameter A, B, basal turn length (BTL) and volume (V) in between sample groups was tested using the two-sided Mann–Whitney-Wilcoxon test with Bonferroni correction. Significant differences are marked by asterisks (*p < 0.05; **p < 0.01; ***p < 0.001; ****p < 0.0001). Data points within boxplots were defined as outliers if they exceeded 1.5 times the interquartile range. Linear correlations were evaluated using the Pearson correlation coefficient.

## Results

**Figure 2:**
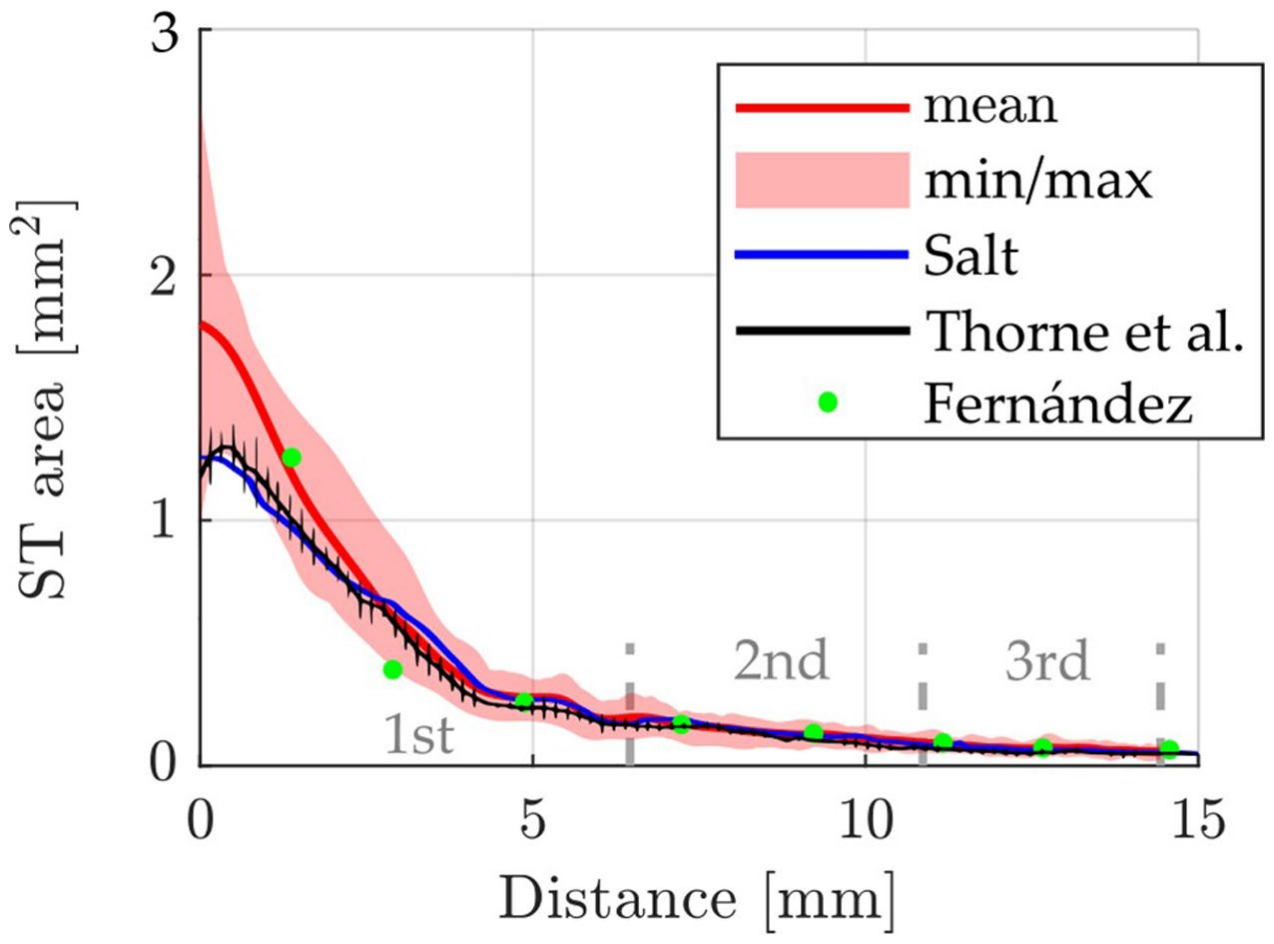
Comparison of the cross-sectional ST area (mean, min and max) over cochlear length of the present study (*n* = 42 ears, from *n* = 26 animals) compared to data reported by Fernandez [*n* = 6 ears, from 3 animals (Fernàndez, 1952)], Thorne et al. [*n* = 3 (Thorne et al., 1999)] and Salt [*n* = 1 (Salt, 2023)]. The 1st, 2nd, and 3rd cochlear turns are indicated.

Initially, the segmented cross-sectional areas of all 42 ears were plotted over the distance along the cochlear spiral, measured along the central axis of the ST. This was done to allow for comparisons to corresponding data proposed by Fernandez (*n* = 6) (Fernàndez, 1952), Thorne (*n* = 3) (Thorne et al., 1999) and Salt (*n* = 1) (Salt, 2023), the result of which is shown in Figure 2. Excellent agreement with Fernandez can be observed after the basal 3 mm of the cochlea while the most basal data point of Fernandez lies noticeably higher than the data of the present cohort. The profile of Salt and Thorne are in the initial range below our mean profile, from 5 mm onwards no difference between the different examinations is recognizable.

**Figure 3:**
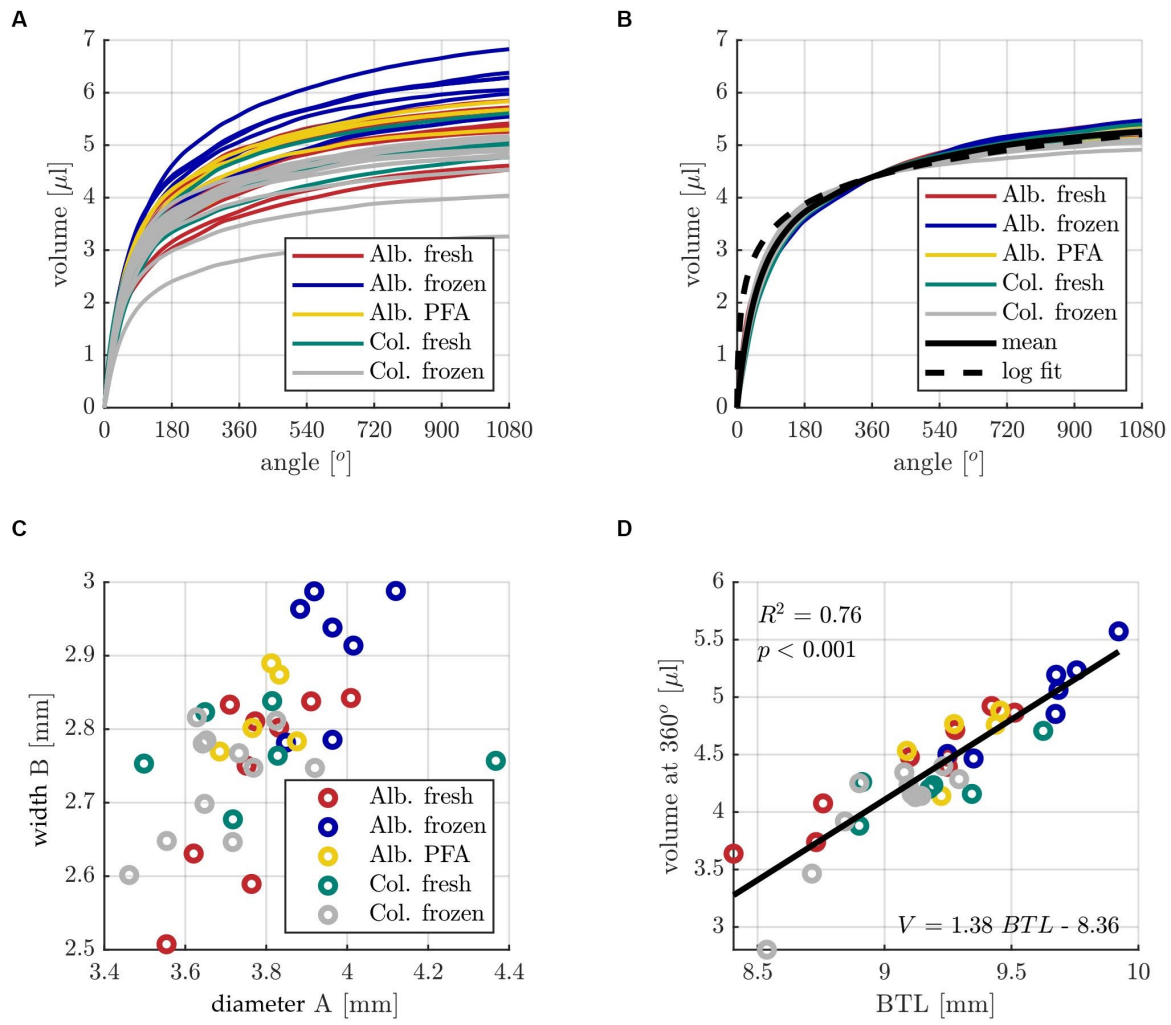
**(A)** Individual profiles of ST volume over cochlear angle for all specimens of the five different groups with color code: dark red Alb. fresh (*n* = 10), indigo Alb. frozen (*n* = 6), yellow Alb. PFA (*n* = 8), teal Col. fresh (*n* = 6) and grey Col. frozen (*n* = 12). **(B)** Individual ST volume profiles normalized to the mean value at 360°, corresponding mean profile and logarithmic fit. **(C)** Distribution of individual ST basal diameters A and widths B. **(D)** Correlation analysis of basal turn length (BTL) computed with the ECA method [with input parameters A and B] (Schurzig et al., 2018b) and the corresponding ST volume at 360°. The constants for the line of best fit determined with the linear regression are V = 1.38 *BTL – 8.36.

Subsequently, the individual volume distributions along the cochlear angle were compared for all samples of the five different groups Alb. fresh (*n* = 10), Alb. frozen (*n* = 6), Alb. PFA (*n* = 8), Col. fresh (*n* = 6) and Col. frozen (*n* = 12). The individual results as well as the mean profiles of the different groups are depicted in Figure 3A. The figure illustrates that all groups show a similar qualitative ST volume profile with a rapid increase within the basal cochlear turn, followed by a much shallower slope within the subsequent two turns. Quantitatively speaking, the average volume at 360° (4.4 ± 0.57 μL) was found to contain 83% of the total ST volume at 1080° (5.3 ± 0.78 μL). The individual ST volumes were therefore normalized to the mean ST volume at 360° and compared to the mean profile of all volumes, which is shown in Figure 3B. Only minimal deviations of the individual, normalized curves to the mean profile can be observed, which further emphasizes the qualitative similarity of the ST volume for all animals independent of the group. Since the shapes of the individual and mean profiles show similarities to a logarithmic function, a logarithmic fit of the mean profile was computed as well. However, Figure 3B demonstrates that the logarithmic fit shows a more rapid increase in volume within the basal 180° and may hence not be very well suited for volume approximations. Nevertheless, the logarithmic fit was employed for ST volume approximations in the following as well with the goal to quantify the resulting deviations. In order to find the origin of the derived quantitative differences in ST volume, A and B value were assessed as a measure of the “footprint” of an individual cochlea. As shown in Figure 3C, A and B values ranged from 3.46–4.37 mm and 2.51–2.99 mm respectively, and although A and B were found to be significantly correlated (*p* < 0.001), the corresponding correlation coefficient of R^2^ = 0.31 shows that the shape of the basal turn can vary quite substantially. That is why, as with the human cochlea, we followed the approach of Schurzig et al. and use the Elliptic-circular approximation (ECA) formula to predict the BTL as a measure of the general cochlea size based on individual measurements of A and B values (Schurzig et al., 2018b). According to this equation, the BTL can be approximated by the following equation:

$$BTL=1.18A+2.69B-\sqrt{0.72\mathrm{AB}}\text{.}$$

The individual BTL values were then correlated with the corresponding ST volumes at 360°, which is shown in Figure 3D and demonstrates that this highly significant correlation explains 76% of the variation in ST volume.

**Figure 4:**
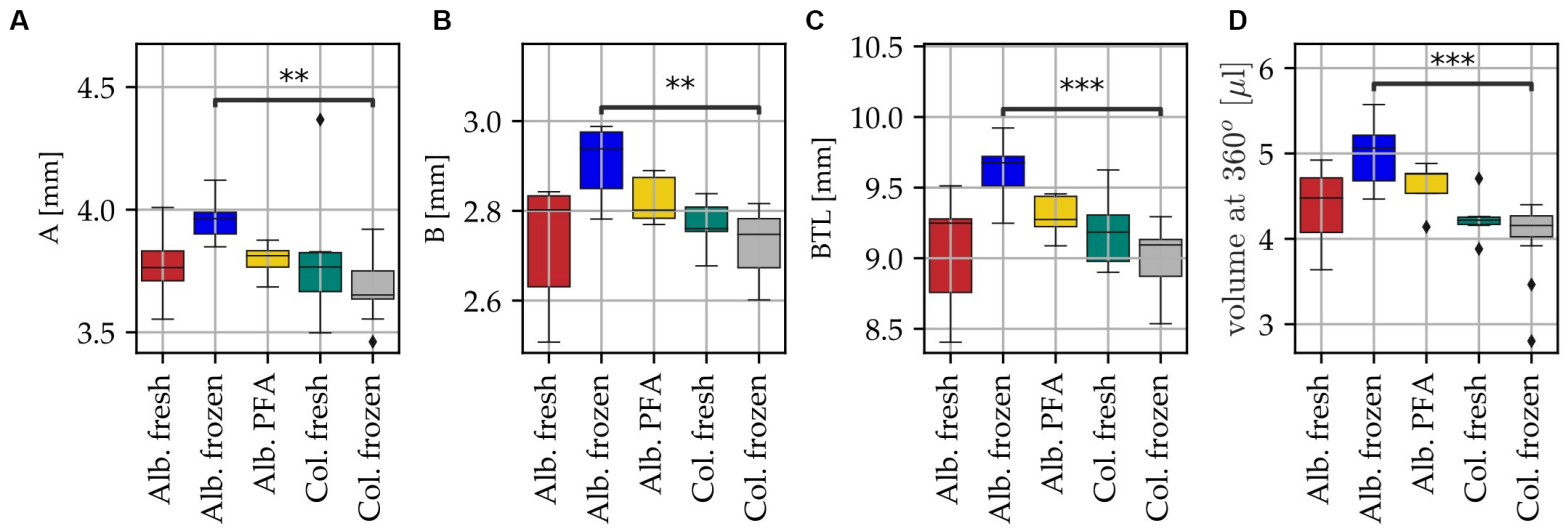
Differences in cochlea anatomy for all specimens of the five different groups with color code: dark red Alb. fresh (*n* = 10), indigo Alb. frozen (*n* = 6), yellow Alb. PFA (*n* = 8), teal Col. fresh (*n* = 6) and grey Col. frozen (*n* = 12). Black diamonds indicate outliers, black bar above boxplot indicates statistically significant differences, ** *p* < 0.01, *** *p* < 0.001. **(A)** Basal diameter A, **(B)** basal width B, **(C)** basal turn length (BTL) computed with the ECA method, with input parameters A and B (Schurzig et al., 2018b) and **(D)** corresponding ST volume at 360°.

It was also investigated if there are any significant differences between the different tissue groups in all the abovementioned cochlear parameters, the results of which are shown in Figure 4. For all three geometric parameters (A, B, BTL value) as well as the volume at 360°, Alb. frozen shows the highest values with medians of A = 3.96 mm, B = 2.94 mm, BTL = 9.68 mm and V = 5.06 μL. Alb. fresh (medians: A = 3.76 mm, B = 2.80 mm, BTL = 9.25 mm and V = 4.48 μL), Alb. PFA (medians: A = 3.81 mm, B = 2.80 mm, BTL = 9.27 mm and V = 4.76 μL) and Col. fresh (medians: A = 3.77 mm, B = 2.76 mm, BTL = 9.18 mm and V = 4.22 μL) are close to each other, with Alb. fresh showing the highest variance for the parameter B and consequently also for BTL. The lowest median values for all parameters are shown by Col. frozen (A = 3.65 mm, B = 2.75 mm, BTL = 9.09 mm and V = 4.16 μL). The scatter in the Col. fresh group is lower in volume than in geometric parameters. The values of Alb. frozen are significantly larger than those of Col. frozen for all four parameters (A: *p* = 0.15*10^−2^; B: *p* = 0.25*10^−2^; BTL: *p* = 0.81*10^−3^; CDL: *p* = 0.58*10^−3^). No significant differences can be found to and among the other groups (see Supplementary Tables S1–S4).

The main aim of the present study was to develop an approach to calculate the volume of perilymph inside the individual guinea pig ST to strengthen pharmacokinetic studies. Based on the results shown in Figure 3, the following methods were derived to approximate the volume of an individual guinea pig ST:

1. Measurement of the A and B value in micro-CT imaging.
2. Calculation of the basal turn length of this specific cochlea using ECA.
3. Approximation of the ST volume at 360° according to Figure 3D (V = 1.38*BTL-8.36).
4. Approximation of the ST volume by
  - Using the mean profile of Figure 3B and neglecting individual anatomical differences (called “mean” in the following).
  - Using the logarithmic function scaled to the individual volume approximation at 360° (called “log” in the following).
  - Using the mean profile scaled to the individual volume approximation at 360° (called “scaling” in the following).

**Figure 5:**
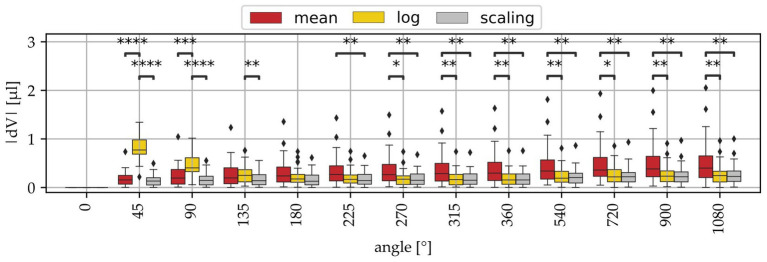
Volume approximation errors (dV) of the 3 derived models (mean, log, scaling) at different angular locations. The mean and log approaches show in part significantly higher deviations than the log model.

In order to evaluate the accuracy of these volume approximation methods, a leave-one-out cross validation was performed on the entire sample set, i.e., the approximation method was re-derived for 37 of the 38 samples and then applied for the 38^th^ cochlea, which was repeated 38 times. Note that although it was shown that there are significant differences in cochlear size in between groups (Figure 4), the present study also demonstrated that the qualitative profile of ST volume along the cochlear angle is very consistent even across sample groups (Figure 3B). That is why the cross validation was conducted across all groups independent of the sample preparation. Figure 5 shows the absolute deviations of these approximations to the measured reference volumes. Large deviation errors can be observed for the logarithmic fit within the basal cochlear region, which was to be expected considering the steep rise of the logarithmic fit function displayed in Figure 3B, Unfortunately, correct approximation of the basal cochlea region is particularly important as it was shown that this region contributes substantially to the overall ST volume (*cf.* Figures 3A,B). The mean model shows significantly larger estimation errors starting at the second half of the basal turn, and the scaling approach yields the lowest estimation errors of all three approaches. However, median estimation errors stay below 0.5 μL for both the mean and scaling models.

**Figure 6:**
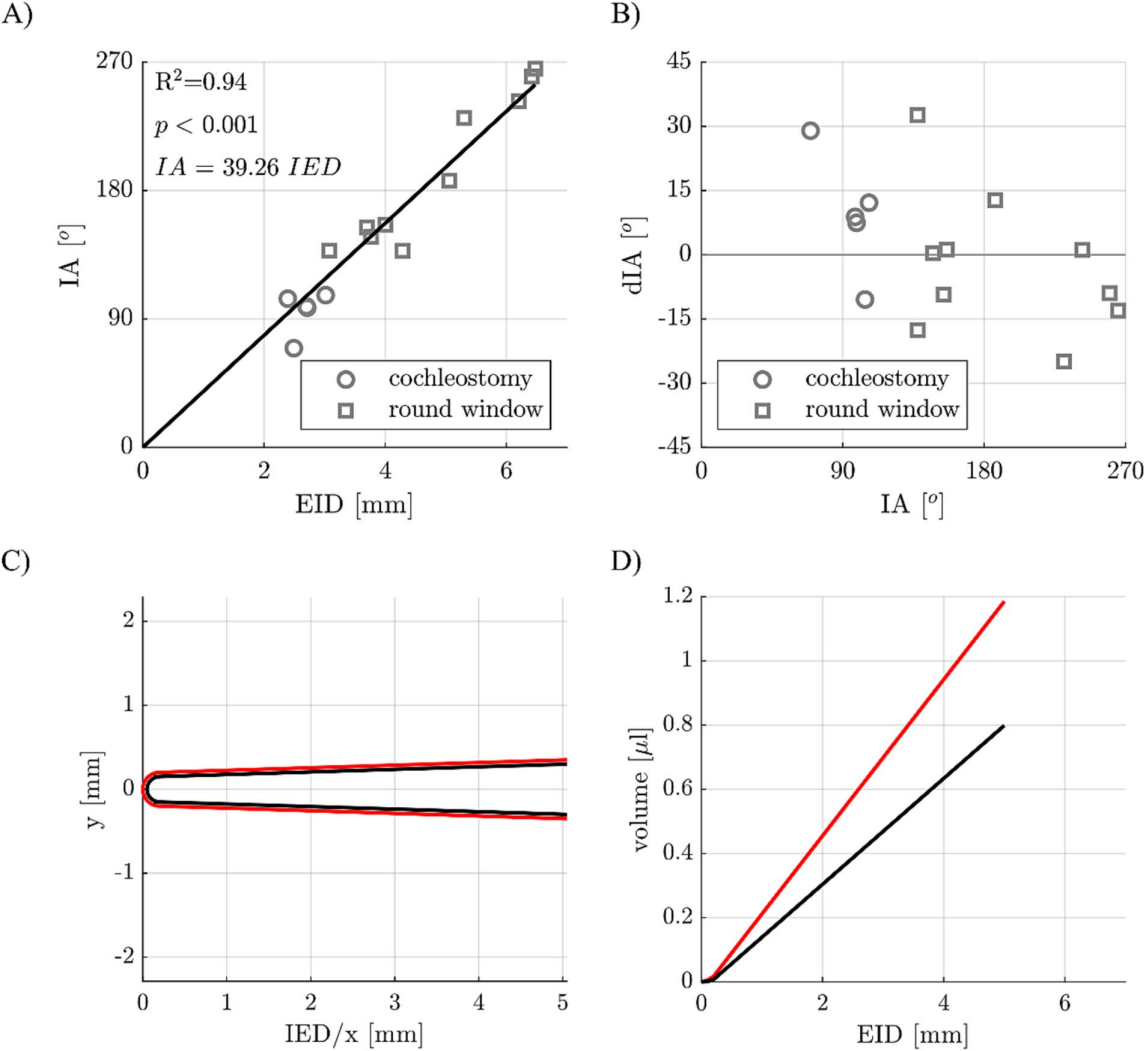
**(A)** Correlation of electrode insertion depth (EID) and insertion angle (IA) for different implant insertion sites (cochleostomy and round window approach). **(B)** Deviations of predicted and postoperatively measured IAs. **(C)** Cross-sectional view of a guinea pig CI array (in black) with a 50 μm coating (in red). **(D)** CI array volume of array alone (black) and with the 50 μm coating (red) dependent on the EID.

In order to derive how deeply an electrode array needs to be inserted into a guinea pig cochlea to reach a specific IA, the IED and IA values measured for round window and cochleostomy CI insertion within the 15 implanted cochleae were correlated. Figure 6A shows the result of this correlation, with a highly significant dependency of IA on IED explaining 94% of variations in IA. An additional leave-one-out cross validation was conducted to investigate whether this correlation can be used for predictions, i.e., the correlation of IA and IED was recomputed for 14 of the 15 samples and then used to predict the IA of the 15^th^ sample, which was repeated 15 times. Deviations dIA of predicted and measured IA of the 15 samples are shown in Figure 6B as a Bland–Altman plot, with a median deviation of 1.1° and a median absolute deviation of 12.6°.

Finally, a virtual model of a guinea pig CI electrode array was created (Figure 6C), which consists of the electrode body (in black) as well as a coating (in red). Using the dimensions of this electrode array (example: apical diameter 0.3 mm, basal diameter 0.6 mm, length 5 mm) in combination with a specific coating layer thickness (example: 50 μm), the volumes of the array alone and the array with the coating can be computed based on the IED, which is shown in Figure 6D.

**Figure 7:**
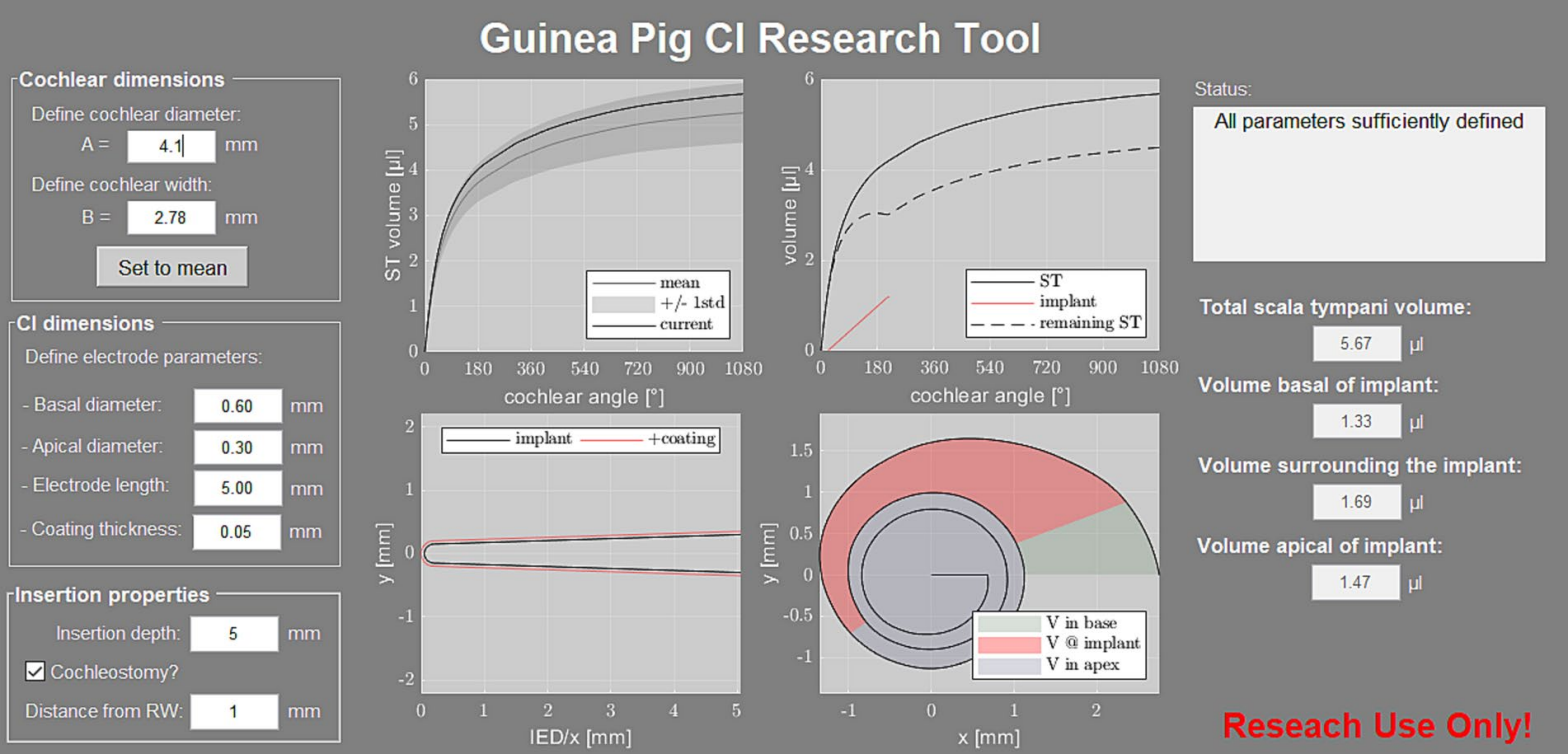
Screenshot of the developed software tool. On the left, the user can define the “cochlear dimensions” for computation of the individual ST volume, which is displayed relative to the mean profile ±1 SD of the present study (top left graph). The tool also requires the “CI dimensions” and “Insertion properties,” based on which the array model is generated (bottom left graph) and the remaining perilymph volume inside the ST is computed (top and bottom right graphs). The corresponding volumes are displayed in the right column of the user interface.

Based on the abovementioned results, a software tool was developed to estimate the ST volume of individual guinea pig specimens. The individual volume is computed based on A and B value of the individual specimen and displayed for the first 3 turns (Figure 7, top left graph). The tool also allows for generating volumetric models of CI electrode arrays including pharmaceutical coatings (Figure 7, bottom left graph) and computes the volumetric properties inside the ST after user-defined array insertion (Figure 7, top and bottom right graphs). The latter includes the computation of the remaining perilymph volume within the three regions relative to the inserted electrode array, i.e., basally of the implant (in case of a cochleostomy), surrounding the implant and apically of the implant. The Matlab based software tool is freely available on our website for download and use.^2^

## Discussion

Many anatomical studies on human cochleae with large numbers of cases have been performed, showing that cochlear anatomy has high variability and that individual differences must be taken into account when selecting electrode type, insertion depth and more (Würfel et al., 2014; Meng et al., 2016; Timm et al., 2018; Schurzig et al., 2018c; Dhanasingh et al., 2021). Since guinea pigs are the favored animal model for testing inner ear pharmacotherapies, we were interested in possible anatomical variations in this species, which can lead to different drug-volume-concentrations and may affect drug therapy outcomes. Our data show that there is also high variability in cochlear anatomy in the guinea pig, leading to differences in ST volume, which should be taken into account when planning and conducting drug trials in the guinea pig animal model.

One of the first studies to calculate the ST volume from cross section size was performed by Thorne et al. (1999) and their determined mean ST volume of 4.7 μL has been considered as a standard value since then. However, only seven pigmented guinea pigs were analyzed in that study and only in 3 of them the ST volume was calculated. In the present study, detailed analyses of the ST volume generated in 26 guinea pigs (*n* = 42 cochleae) are reported, including consideration of strain and postmortem processing. One aim of the present study was to examine whether the factors of strain and *post mortem* processing have an impact on the measurement of specific cochlear parameters. Our study shows a change in cross-sectional area of the ST along the cochlear spiral, which is comparable to previous studies (Figure 2; Fernàndez, 1952; Thorne et al., 1999; Salt, 2002; Salt, 2023). Unfortunately, the comparability to those studies is limited. This is due to the small number of cochleae investigated within those studies, the differences in postmortem processing of the tissue and usage of histological preparations instead of micro-CT images for analysis. Furthermore, only 9 statistical measurement points are available in Fernàndez (1952), limiting comparability to only 3 points per cochlear turn. Also, it must be noted that the studies of Thorne et al. (1999), Salt (2023), and Salt (2002) used different calculations to determine the ST volume from ST area than in the present study and thus a comparison of ST area, but not the total ST volume, is possible. In general, the volume profile between the literature values and ours is very similar, with Thorne et al. and Salt values being below our mean up to a cochlear length of 3 mm, but in good agreement beyond 3 mm. Interestingly, both the Salt study and our results show a plateau in ST area at about 5 mm distance from the round window. This plateau, which is a temporary stop of the decrease in ST area, is likely the origin of the slight bumps in ST volume which can be observed within this area, i.e., between 270–360° (*cf.* Figures 3A,B). After this plateau, the cross-sectional area drops again prior to the beginning of the second cochlear turn. Studies on the development of the cochlea show that the cochlear lumen develops from the base to the apex and around pre-existing neural structures (Pietsch et al., 2017). The drop in ST area at the end of the basal cochlear turn may hence be owed to spatial constraints during development: at the beginning of the second turn, the ST develops in very close proximity to the scala vestibuli of the basal turn which substantially limits the available space for growth.

The most common method of determining ST volume is manual segmentation of cochlear structures from imaging data. It is obvious that the quality of the measurement is hence directly related to the quality of the images. In our study, 42 cochleae were examined, of which 20 segmentations from the 2nd turn were described as difficult because the tissue structures were hard to identify. Direct comparison of the groups revealed a noticeably larger variability in all investigated dimensions for the Alb. fresh group compared to the Col. fresh group. Furthermore, we found significant differences between Alb. frozen and Col. frozen samples, with the volume and geometric parameters of the Alb. frozen samples being significantly larger. A possible explanation for this is the more difficult delineation of the ST in the Alb. frozen samples due to the absence of melanocytes in the stria, affecting the change in Hounsfield units when progressing bone to the intrascalar spaces and potentially making the ST appear larger in the resulting segmentations. This in combination with the poorer quality of the thawed samples compared to the fresh albino samples may have led to an overestimation of the ST area. No significant differences were found to and between the other groups. Data were only obtained from PFA fixed albino animals, not from colored animals. Since fresh albino tissue and colored tissue did not differ in volume but frozen tissue does, another group with fixed colored animals could have provided additional information on ST volume differences between albino and colored animals. Nevertheless, based on the high variability in ST volume we report here, we claim that for future studies on pharmacotherapeutic applications to the inner ear, the scalar volume should be calculated for the specific method applied in the respective experimental setting, i.e., each laboratory should calculate the drug amount to be applied based on the guinea pig breed used and specific histological methods applied.

To examine guinea pig cochlear variability in more detail, we determined the volume profile along the entire cochlear spiral instead of overall volumetric measures. In general, we saw a similar volume profile within each group, with attainment of 83% of the total volume already in the basal cochlear turn. For comparison with human studies, the relative standard deviation (RSD) was calculated. Our data showed a volume variability of 14.7% RSD with a total mean volume of 5.3 ± 0.78 μL, comparable studies in humans showed a volume variability between 6.3% RSD (Hussain et al., 2023) to 20% RSD (Dhanasingh et al., 2021). To determine the cochlear “footprint,” the parameters A and B were assessed. In human medicine these parameters are now used as a standard to describe cochlear anatomy and serve as a basis for selecting electrode type and insertion depth (Escudè et al., 2006; Würfel et al., 2014; Schurzig et al., 2016, 2018b; Avallone et al., 2021). Based on the present data, there is a 5.3% RSD observed for parameter A, with a mean value of 3.8 ± 0.2 mm. Similarly, parameter B shows a deviation of 3.6% RSD from the mean, with a mean value of 2.8 ± 0.1 mm. These deviations are comparable to previous human studies, such as Pietsch et al. (2017) with 4.4% RSD, Escudè et al. (2006) with 5.3% RSD, Dhanasingh et al. (2021) with 5.6% RSD, and Hussain et al. (2023) with 6.4% RSD, all for A value. Furthermore, for B value, Hussain et al. (2023) reported a deviation of 3.7% RSD, Pietsch et al. (2017) reported 4.4% RSD, and Dhanasingh et al. (2021) reported a 6.0% RSD. Thus, our data show similar variability in cochlear anatomy and ST volume, A and B value as in humans.

The parameters A and B can be used to determine the BTL. This is applied in clinical practice to determine the insertion depth and angle (Timm et al., 2018; Schurzig et al., 2018a; Avallone et al., 2021). This approach was used to generate the “Guinea Pig CI Research Tool,” a software tool that creates an individual volumetric profile of a guinea pig ST based on the respective values of A and B. This individualized reconstruction of the ST can be considered as an influencing parameter when analyzing animal model pharmacotherapy data. The advantage of this tool is that it is no longer necessary to manually segment the entire cochlea, which was difficult in 20 out of 44 cases in our study, especially from the second turn onward. Instead, only the two easily distinguishable parameters A and B have to be measured. Our model has been tested using leave-one-out cross-validation and is resilient to difficult measurements.

A number of studies focused already on inner ear pharmacokinetics and ST volume. Among them, the work by Alec Salt and colleagues is the most cited one. They published a freely accessible tool named FluidSim (Salt, 2002; Salt, 2023) to calculate drug distribution within the inner ear for 6 different species, including humans and guinea pigs. Using FluidSim the delivery of drugs can be simulated using different methods (intratympanic application, round window niche implant, intracochlear injection, eluting CI and systemic application). Furthermore, the active ingredient can also be freely selected, such that the molecular property, affecting the distribution, is taken into account. The drug concentration is now calculated over time and distance for various selectable cochlear structures, such as the ST volume. However, a fixed ST volume is assumed as the basis (default volume of the guinea pig from the model is shown in Figure 2). As described before, we could show that the volume has a variability with of 14.7% RSD, A value 5.3% RSD and B value 3.6% RSD. These individual variabilities may cause large differences in active agent studies on pharmacogenetics and –dynamics since the variance in cochlear geometry is not taken into account in previous models.

An increasing focus in fighting cochlear implantation related pathologies such as fibrosis and neuronal degeneration is local pharmacotherapy via a single application of an active ingredient by means of a syringe (Paasche et al., 2006), with the aid of a catheter (Prenzler et al., 2018) or by CI based drug delivery (Scheper et al., 2017, 2019; Dhanasingh et al., 2021; Manrique-Huarte et al., 2021; Behrends et al., 2023). In preoperative planning for CI, it is already common practice to take the patients individual anatomy into account. When performing guinea pig studies on CI based drug delivery, this is not done yet. Since our study proves a variability of 14.7% RSD between individual guinea pigs, it is obvious that the individual ST volume needs to be considered as well. To plan and analyze appropriate animal experiments it is necessary to know the drug concentration at different locations of the cochlea, and thus how large the ST volume and the displaced volume by the CI electrode will be.

We can now show for the first time a model that cannot only calculate the individual ST volume of guinea pigs based on two anatomical measures, but also additionally considers the volume of the CI electrode. Furthermore, it can take into account whether the electrode has been coated with active substance and, in addition to the total ST volume, displays the volume surrounding the electrode, as well as an estimate of the volume basal and apical to the implant. This information can be of great help in planning drug studies and may help explain differences in studies already performed. We recommend using the developed and herewith published software tool (see Footnote 2) for planning and conducting future studies on pharmacotherapy of the inner ear using guinea pigs as animal model. Using the software tool, no time-consuming manual segmentation on high resolution images is needed. By routine pre-surgical micro-CT imaging of the cochlea applying low and short x-ray exposition the A and B values can be determined easily. No contrast agent is necessary to measure A and B values. A description on how to measure A and B is provided within the handbook of the software tool, which can be downloaded using the same link. The values can subsequently be used to calculate the volume of the ST by entering them into the software tool. The volume should finally be taken into account when planning the drug delivering strategy. Individualized drug concentrations can be incorporated into delivery matrices, or individually adapted drug concentrations can be applied as injections into the individually sized ST.

It should be noted, however, that the model is currently only validated for the non-commercial MED-EL guinea pig electrodes manufactured specifically for these kinds of experiments, for other electrode types this has yet to be done. The method for calculating ST volume using geometric parameters, A and B value, can also be adapted for other species, the model must be reviewed and adjusted if necessary. We believe that an invaluable resource for pharmacokinetics research would be the integration of Salt’s highly regarded and continuously enhanced FluidSim model (Salt, 2002; Salt, 2023). By combining this established model with our newly developed tool, which emphasizes the individual characteristics of cochlear geometry and its impact on ST volume, we can greatly enhance our understanding in this field. This can lead to a reduction in the number of animals used in experiments due to the improved transferability of results from animal studies to humans.

## Data availability statement

The raw data supporting the conclusions of this article will be made available by the authors, without undue reservation.

## Ethics statement

The animal study was approved by Lower Saxony State Office for Consumer Protection and Food Safety (LAVES), Oldenburg, Germany, regional council registration numbers 21/3703 and 17/2396. The study was conducted in accordance with the local legislation and institutional requirements.

## Author contributions

MG: Writing – original draft, Data curation, Formal analysis, Investigation, Validation, Visualization. KM: Data curation, Writing – review & editing, Formal analysis, Investigation, Visualization. TL: Writing – review & editing, Funding acquisition, Resources, Supervision. VS: Writing – review & editing, Conceptualization, Data curation, Funding acquisition, Investigation, Project administration, Resources, Supervision. DS: Writing – review & editing, Conceptualization, Data curation, Formal analysis, Investigation, Methodology, Project administration, Resources, Software, Validation, Visualization.

## Footnotes

^1^ www.slicer.org

^2^ https://vianna.uber.space/01_workgroups/scheper/guinea%20pig%20volume%20model.html#fnref:1
