# Supplementary material for: Optimization of pharmacological interventions in the guinea pig animal model—a new approach to calculate the perilymph volume of the scala tympani
Source: Front Neurosci. 2023 Dec 15;17:1297046. doi: 10.3389/fnins.2023.1297046 (PMC10754993; doi:10.3389/fnins.2023.1297046)
Supplement: Supplementary file 1 [file Data_Sheet_1.PDF]

Supplementary Material to

Optimization of pharmacological interventions in the guinea pig animal model – a new approach to calculate the perilymph volume of the scala tympani

Marleen Grzybowski[1,2], Kathrin Malfeld[1,3], Thomas Lenarz[1,2,3], Verena Scheper[1,3], Daniel Schurzig[1,4]

[1] Dept. of Otorhinolaryngology, Head and Neck Surgery, Hannover Medical School, Hannover, Germany

[2] German Hearing Center Hannover, Hannover Medical School, Hannover, Germany

[3] Center for Biomedical Engineering, Implant Research and Development (NIFE), Hannover Medical School, Hannover, Germany

[4] MED-EL Research Center, Hannover, Germany

	Alb. fresh	Alb. frozen	Alb. PFA	Col. fresh	Col. frozen
**Alb. fresh**	-	1.107e-01	1.000	1.0000	1.000
**Alb. frozen**	1.107e-01	-	9.366e-02	5.378e-01	1.525e-02
**Alb. PFA**	1.000	9.366e-02	-	1.000	8.926e-01
**Col. fresh**	1.000	5.378e-01	1.000	-	1.000
**Col. frozen**	1.000	1.525e-02	8.926e-01	1.000	-

Tab. S1: p-values of the two-sided Mann-Whitney-Wilcoxon test with Bonferroni correction for the basal diameter A in between guinea pig groups. Significant differences are highlighted in red

	Alb. fresh	Alb. frozen	Alb. PFA	Col. fresh	Col. frozen
**Alb. fresh**	-	2.622e-01	1.000	1.000	1.000
**Alb. frozen**	2.622e-01	-	1.000	1.842e-01	2.802e-02
**Alb. PFA**	1.000	1.000	-	1.000	4.143e-01
**Col. fresh**	1.000	1.842e-01	1.000	-	1.000
**Col. frozen**	1.000	2.802e-02	4.143e-01	1.000	-

Tab. S2: p-values of the two-sided Mann-Whitney-Wilcoxon test with Bonferroni correction for the basal width B in between guinea pig groups. Significant differences are highlighted in red

	Alb. fresh	Alb. frozen	Alb. PFA	Col. fresh	Col. frozen
**Alb. fresh**	-	1.107e-01	1.000	1.000	1.000
**Alb. frozen**	1.107e-01	-	5.132e-01	1.242e-01	8.053e-03
**Alb. PFA**	1.000	5.132e-01	-	1.000	4.143e-01
**Col. fresh**	1.000	1.242e-01	1.000	-	1.000
**Col. frozen**	1.000	8.053e-03	4.143e-01	1.000	-

Tab. S3: p-values of the two-sided Mann-Whitney-Wilcoxon test with Bonferroni correction for the basal turn length BTL in between guinea pig groups. Significant differences are highlighted in red

	Alb. fresh	Alb. frozen	Alb. PFA	Col. fresh	Col. frozen
**Alb. Fresh**	-	2.622e-01	1.000	1.000	9.464e-01
**Alb. Frozen**	2.622e-01	-	1.000	8.221e-02	5.784e-03
**Alb. PFA**	1.000	1.000	-	1.000	2.347e-01
**Col. Fresh**	1.000	8.221e-02	1.000	-	1.000
**Col. Frozen**	9.464e-01	5.784e-03	2.347e-01	1.000	-

Tab. S4: p-values of the two-sided Mann-Whitney-Wilcoxon test with Bonferroni correction for the ST volume V in between guinea pig groups. Significant differences are highlighted in red
